# When policy-makers have your back: the Kerala experience with state-wide antimicrobial resistance mitigation efforts

**DOI:** 10.1017/ash.2025.10060

**Published:** 2025-07-11

**Authors:** Aravind Reghukumar, Rajalakshmi Ananthanarayanan, K.V. Nandakumar, Sivaprasad P.S., Manjusree S., Jyothi R., Heera Hassan, Ajan Maheswaran Jaya, H. Sreenadh, R. Nikhilesh Menon

**Affiliations:** 1 Department of Infectious Diseases, Government Medical College, Thiruvananthapuram, Kerala, India; 2 Infectious Diseases, KIMSHEALTH, Trivandrum, Kerala, India; 3 Additional Director of Health Services [Medical], State Program Officer, AMR Health Services, Government of Kerala, Trivandrum, Kerala, India; 4 National Health Mission, Government of Kerala, Trivandrum, Kerala, India; 5 Department of Microbiology, Government Medical College, Thiruvananthapuram, Kerala, India; 6 Health Services-Medical and Hospital Administration, Government of Kerala, Thiruvananthapuram, Kerala, India; 7 Department of Health Services, Family Health Centre Noolpuzha, Wayanad, Kerala, India; 8 Department of Health Services, One Health and AARDRAM mission, Government of Kerala, Thiruvananthapuram, Kerala, India

## Background

Antimicrobial resistance (AMR) has been recognized as one of the biggest public health challenges by the World Health Organisation (WHO). On September 21, 2016, the United Nations (UN) General Assembly met to discuss AMR.^1^ Although AMR is a natural phenomenon, it is propagated by misuse of antimicrobial medicines, inadequate or nonexistent programs for infection prevention and control (IPC), poor-quality medicines, weak laboratory capacity, inadequate surveillance, and insufficient regulation of antimicrobial drugs. As AMR threatens the very core of modern medicine, in May 2015, the World Health Assembly adopted a global action plan on AMR.^2,3^ During the 79th UN General Assembly meeting on AMR, held on September 26 2024, global leaders approved a political declaration related to AMR. These included committing to a clear set of targets and actions, like reducing the estimated 4.95 million human deaths associated with bacterial AMR annually by 10% by 2030.^4^ The declaration calls for sustainable national financing and USD100 million in catalytic funding to support funded national action plans on AMR. The declaration also sets an ambitious target that at least 70% of antibiotics used for human health globally should belong to the WHO Access group, 100% of countries having basic water, sanitation, hygiene, and waste management services in all health care facilities, and 90% of countries meeting WHO’s minimum requirements for IPC program by 2030. AMR mitigation efforts require a whole-of-society approach with strong political will and commitment to ensure that all stakeholders work in unison and implement local, context-specific solutions. It is in this context that AMR mitigation efforts taken by Kerala, a state in South India known for its high literacy rate, are discussed here. The AMR program in Kerala is called the Kerala Antimicrobial Resistance Strategic Action Plan (KARSAP).^5^ The highlight of the program is the policymakers of the state considering AMR as a crisis, taking the lead, and supporting the technical experts for the execution. Another unique aspect is the measures taken for AMR mitigation, which is based on participatory antimicrobial stewardship (AMS), whereby, through district and block-level AMR committees and public participation, a whole-of-society approach is ensured. Government of Kerala (GoK) initiatives toward addressing the AMR crisis are appreciated, nationally and internationally.

## KARSAP (Kerala antimicrobial resistance strategic action plan)

**Figure 1.**
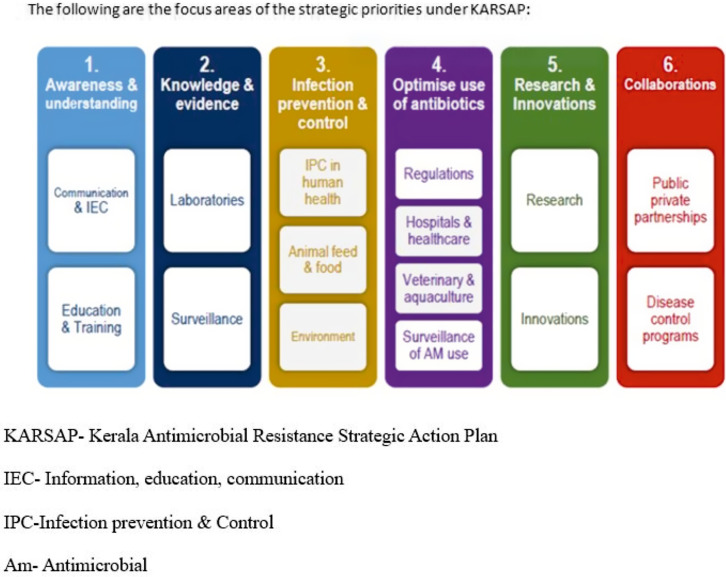
Strategic priorities under Kerala Antimicrobial Resistance Strategic Action Plan.

Global Action Plan on AMR was released in 2015.^3^ Indian National Action Plan on AMR (NAP-AMR) was released in 2017.^6^ In 2018, Kerala became the first state in India to release a sub-national action plan called KARSAP, which is conceptualized and implemented on the One Health platform and was jointly drafted by the departments of Health, Animal Husbandry, Environment, Fisheries and Agriculture.^7^ The 4 main objectives of KARSAP are to create AMR surveillance systems in Kerala, develop multisectoral strategies to reduce AMR, periodically review the AMR surveillance data to plan future actions, and incorporate them into prevention and management strategies. In line with the Global and National Action Plan, KARSAP has six strategic priorities as shown in Figure 1. In 2022, a time-sensitive monitoring and evaluation framework was conceptualized to coordinate and augment AMR mitigation strategies in the human side, animal husbandry, fisheries, aquaculture, environment, and other sectors. KARSAP targets to be attained in a specified time frame on the human side are depicted in Table 1.^8^

**Table 1.** Kerala Antimicrobial Resistance Strategic Action Plan targets to be attained and time frame

Target	1 year	3 years	5 years
District level AMR committees to be established	√		
Block level AMR committees to be established	√		
Awareness generation on AMR among school students	√		
AMR to be included in school curriculum		√	
Hub and spoke model of AMR surveillance in all districts		√	
Accreditation for all tertiary care institutions		√	
Accreditation for all primary and secondary care institutions			√
Zero effluent discharge policy to be implemented in all hospitals, farms, pharma industry			√
Expansion of PROUD° to all districts in Kerala		√	
Converting all PHCs to Antibiotic smart PHCs^1^	√		
WHO AWaRe Target to be attained		√	
Antimicrobial utilization metrics calculated in all hospitals		√	
Achieve 50% reduction in carbapenem resistant g infections		√	

°PROUD- program for removal of unused drugs.

1 PHCs-Primary Health Centers

The one-year, three-year, and five-year time-sensitive targets under KARSAP 1.0 (2018–2024) focused more on process indicators related to the containment of AMR, aligning with the six strategic priorities. For KARSAP 1.0, most funding was utilized to establish the AMR surveillance network (KARSNET) and to strengthen the microbiologic diagnostic capacities in secondary care hospitals. KARSAP 2.0 is set to be launched by 2025, with one of its objectives being a one-health-based cost-effectiveness analysis. Funding for IEC posters, digital modules, and public awareness campaigns comes from budgetary provisions allocated for quality improvement initiatives at the hospital and LSG (Local self-government) levels. To establish KARSNET, assistance was obtained from the NCDC (National Centre for Disease Control) and the WHO, India.

## Strategic Priority 1- Awareness and Understanding

### Participatory Antimicrobial Stewardship - Antibiotic Literate Kerala Campaign (ALKC)^9^

**Figure 2.**
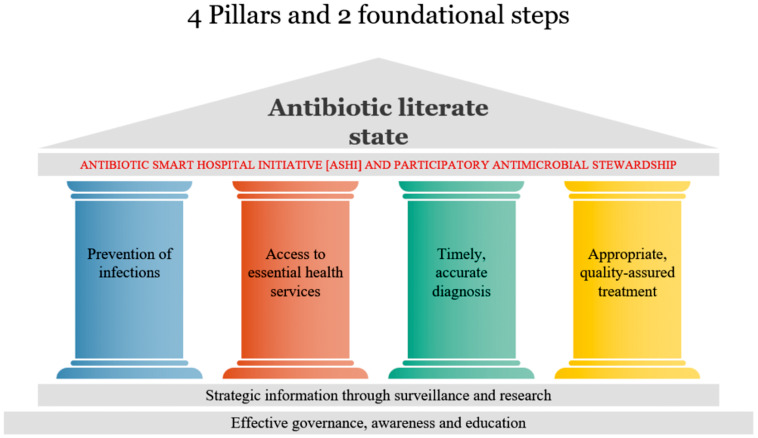
4 Pillars and 2 foundational steps..

Fostering patient and public engagement (PPE) to mitigate AMR is termed participatory antimicrobial stewardship (AMS). This puts people and their needs at the center of the AMR response. PPE has been adopted as an important target only in a few national AMR policies. The importance of having a people-centered approach to tackle AMR was acknowledged by WHO guidelines issued on October 19, 2023.^10,11^ These core interventions are based on four pillars and two foundational steps critical to overcoming barriers faced by people and health systems in addressing AMR.^10^ The four pillars are: (1) infection prevention; (2) access to essential health services; (3) timely, accurate diagnosis; and (4) appropriate, quality-assured treatment. The two foundational steps that support the pillars are (1) effective governance, awareness, and education; and (2) strategic information through surveillance and research (Figure 2).

Under KARSAP, ALKC was launched in 2019 to make all citizens of Kerala aware of the judicious use of antibiotics in all sectors through a participatory AMS program.^9^ Due to the COVID-19 pandemic, activities of ALKC came to a standstill in 2020 and 2021. From 2022 onwards, public measures under ALKC have gained momentum. The broad objectives envisaged under AKLC are universal awareness about the importance of having access to antibiotic-free food and water, consuming antibiotics only on a doctor’s prescription, and safely disposing of unused or expired antibiotics. Kerala started a unique campaign termed Program on Removal of Unused Drugs (PROUD) to address this.^12^

To make Kerala an antibiotic-literate state, several initiatives have been launched by the GoK, which include the preparation of online training modules on AMS in the regional language (Malayalam) for the public, farmers, and students. This was released as an AMR information, education, communication (IEC) dossier for ready reference.^8^

### Decentralized approach to implementation of antibiotic literate Kerala campaign

Over the last two decades, Kerala adopted decentralization in public governance, which is recognized globally as one of the most significant institutional reforms.^13^ In similar lines, a decentralized approach was planned for ALKC and implemented through the creation of district and block-level AMR committees. In 2023, Kerala achieved a significant milestone by becoming the first state in India to establish AMR committees in all districts and 190 health blocks.^8^ The AMR committees at the district and block levels are truly One Health-based. Monitoring and evaluation framework and standard operating procedures for block and district AMR committees have been issued as Government Orders (GO) by the GoK. During World AMR Awareness Week activities in 2023, IEC activities were conducted in all districts in Kerala. Family Health Centre (FHC) Kakodi was declared the first antibiotic-smart hospital in Kerala state. On January 1, 2024, Ozlapathy FHC in Palakkad district was declared an antibiotic-smart hospital.^8^

### Ongoing Plans- Deliverables under Strategic Priority One (short-term goals)^12^

- To make all panchayats/ municipalities/ towns antibiotic literate.
- The community volunteers are being trained to identify AMR hotspots in each panchayat, like poultry farms, dairy farms, aquaculture, butcher shops, pharmaceutical industries, hospital effluents, etc. All the identified AMR hotspots will be tagged geospatially, and focused IEC interventions will be delivered. ^15^

An example of a people-centered approach to AMR is the antibiotic literacy campaign being conducted by the Ernakulam district in Kerala. As part of ALKC, 2287 ASHA (Accredited Social Health Activist) workers have been trained in the district to create awareness of AMR among the public.^16^ As of now, more than two lakh households have been covered in the district. Urban, rural as well as migrant populations (referred to as guest workers in Kerala) are addressed as part of this campaign.^16^

## Strategic priority 2—knowledge and evidence

KARS-NET (Kerala Antimicrobial Resistance Surveillance Network) was established in 2018 with the help of the WHO country office for India and the National Centre for Disease Control (NCDC).^8^ In Kerala, the health delivery is broadly divided into three categories: Primary (Family Health Centres), secondary and tertiary care centers. Before the advent of KARSAP, culture-guided antimicrobial therapy was being practised only in tertiary care centers. To get representative data from private hospitals and primary and secondary care institutions, KARS-NET has expanded to include 59 satellite centers (tertiary care) and 185 spokes (secondary and primary care hospitals) with the government medical college (GMC) Thiruvananthapuram as the focal point. In 2022, Kerala became India’s first state to publish its antibiogram, which reflects the antimicrobial susceptibility pattern among priority pathogens in tertiary care centers.^8^ In 2019, in Ernakulam district, a hub and spoke model of AMR surveillance and stewardship was initiated. Through this, culture-guided therapy in secondary care centers was initiated. In 2023, Kerala published the first district antibiogram for Ernakulam district, which reflected the susceptibility trends in secondary care institutions; carbapenem resistance is very rare in secondary tier hospitals.^8^ Further, district antibiograms were initiated in 13 out of 14 districts in Kerala. It was based on these inferences that tier-specific antibiotic guidelines were issued to enable the physician to select the right empiric antibiotic based on the existing antibiogram. AWaRe (Access, Watch, Reserve antibiotic groups, by WHO) based culture reporting format was conceptualized and published for the first time globally by GMC Thiruvananthapuram.^17^ This format is used in most AMR hub centers in Kerala as a decision-making AMS nudging tool.

### Future plans- deliverables under strategic priority 2

- Generation of spoke antibiograms (stratified community antibiogram) in all districts-short term goal (3 years)
- Generation of Antibiogram for animal husbandry department- short-term goal (3 years)

### Antibiotic smart hospitals: a pragmatic approach to tier based antimicrobial stewardship

The GoK, as part of KARSAP, took a significant step in combating AMR by launching the Antibiotic Smart Hospital Initiative (ASHI).^15,9^ Both public and private hospitals that meet the criteria for becoming an Antibiotic Smart Hospital (ASH) are entitled to receive a certificate and an emblem, which can be published and promoted in the public domain, enhancing the hospital’s credibility, public recognition, peer recognition and accreditation process. The media publicity surrounding the declaration of antibiotic smart hospitals encourages local self-government institutions (LSGI) to actively support the hospital in their efforts to become antibiotic smart. By the third quarter of 2025, all the hospitals (Public and Private) and LSGI have been directed by the Government to color code their institutions per the number of criteria achieved. Based on the present color code, they have been directed to frame a micro action plan to improve their color grade within 6 months. Antibiotic smart criteria have been formulated for Dental hospitals as well. The criteria for becoming ASH differ for primary, secondary, and tertiary care institutions based on the patient profile they cater to and available resources. Table 2 highlights the criteria to be satisfied by hospitals in each tier to be declared antibiotic-smart.

**Table 2.** Criteria for antibiotic smart hospitals

	PRIMARY CARE LEVEL	SECONDARY CARE LEVEL	TERTIARY CARE LEVEL
Display of Posters on AMR in Malayalam	√	√	√
All healthcare workers and students fully trained in AMR and IPC	√	√	√
Prescription audit quarterly	√	√	√
Antibiotics utilization metrics calculation quarterly by pharmacy	√	√	√
AWaRe metrics- ACCESS usage in OPD	95%	90%	85%
Functional HICC and AMSP committee	√	√	√
IEC for public fortnighly	√	√	√
Posters on AWaRe classification in prescribing areas	√	√	√
Certified by	NQAS (or within 1 year)	NQAS/KAYAKALP /NABH(or within 2 years)	NQAS/KAYAKALP /NABH(or within 2 years)
Implementation of PROUD program	√	√	√
Surgical safety checklist in surgical specialities		√	√
Prospective audit and feedback for Reserve antibiotics by AMSP team		√	√
Low hanging fruit model of AMSP		√	√
HAI surveillance		√	√
STPs (Sewage Treatment Plants)/ ETP (Effluent Treatment Plant) according to the guidelines from pollution control board		√	√

IEC- Information, education, communication, HICC-Hospital Infection Control Committee, AMSP- Antimicrobial Stewardship Program, NQAS- National Quality Assurance Standards, NABH- National Accreditation Board for Hospitals & Healthcare providers, HAI- Healthcare associated infection.

Currently, 2 FHCs in Kerala (Kakkody and Ozhalapathy) have become antibiotic-smart hospitals. All other hospitals are expected to become antibiotic-smart in a time-sensitive manner. A color grid based on a scoring system has been designed to assess the number of criteria each hospital in the state has achieved in its progress toward attaining antibiotic-smart status.

### Other AMS and IPC initiatives under KARSAP

- Operation AMRITH - A toll-free number is provided for the public to report if antibiotics are dispensed without a prescription. Antibiotic dispensing without a prescription will result in the cancelation of the pharmacist’s license.^18^ Government of India, as an amendment to the Drugs and Cosmetics rule of 1945, passed the Schedule H1 rule [stipulates retail dispensing of all antibiotics only against a valid prescription] in 2011. But due to implementation hurdles in many states, the revised Schedule H1 rule was introduced in 2013. Under the revised Schedule H1 rule, a prescription is required only for second-line and third-line antibiotics. But Kerala, as part of KARSAP, implemented the stringent Schedule H rule, which any other state in India has not done.
- An AI-based interactive Antibiogram App has been launched with the help of K-DISC (Kerala Development and Innovation Strategic Council).^19^
- State-level formulary restriction with the preferential supply of antibiotics from the Access category to primary care centers is planned.
- Directions have been given to pharmacies and hospitals to ensure that antibiotics are dispensed only in blue cover (Go Blue for AMR) with printed IEC messages as part of patient and public education.^20^
- The State Pollution Control Board has established an AMR research lab, and One Health AMR research workshop is organized annually.
- The Drugs Control Department has launched the ROAR (Rage on Antimicrobial Resistance) initiative.^21^ This campaign focused on addressing the growing problem of antimicrobial resistance, ensuring the responsible and effective use of antibiotics.
- Kerala Drugs Control Department launched Operation Vet-biotic to counter the unrestricted sales of veterinary antibiotics in Kerala. ^22, 23^
- The state IPC program will be launched soon.

**Figure 3.**
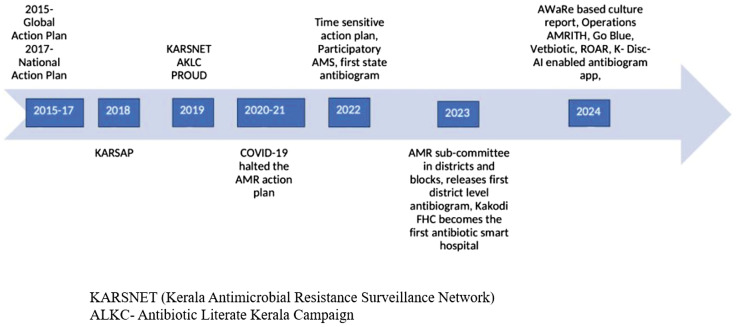
Timeline of Kerala Antimicrobial Resistance Strategic Action Plan initiatives.

The time line of KARSAP initiatives from the inception is depicted in Figure 3.

### Future plans- deliverables under antimicrobial stewardship

- To develop a framework for antibiotic use tracking by pincode (zipcode) - short-term goal, 2 years.
- To develop healthcare tier-based antibiotic guidelines, with a short-term goal of 1 year.
- To incorporate AWaRe color coding matrix into e-health with visual cues to guide prescription- short-term goal, 3 years.^24^
- Promote Antibiotic Smart Hospitals among all private healthcare institutions in a time-sensitive manner. To help implement this, representatives from state chapters of different medical societies like the Indian Medical Association, Indian Paediatrics Association, Clinical Infectious Diseases Society of India, Association of Physicians of India, and the Indian Dental Association have been incorporated into working committee of KARSAP.^25^

### Challenges

KARSAP was the first state-level action plan launched in India. The monitoring and evaluation framework of KARSAP was drafted according to the Global Action Plan on AMR released in 2015, which focused more on process indicators than outcome indicators. Following the implementation of KARSAP, there has been a 30% reduction in antibiotic sales in Kerala (unpublished data). It is too early to determine whether this decrease in sales will translate to a reduction in AMR trends. Another target set by the UN General Assembly High-level meeting on AMR held on September 26, 2024, is to achieve a 10% reduction in AMR-associated deaths by 2030. KARSAP is implemented in such a way as to assist the state in achieving this target by 2030 by optimizing IPC practices and antimicrobial stewardship.

A one-size-fits-all model for AMR mitigation efforts will be suboptimal as the challenges are heterogeneous and locality-specific. Hence, customized templates are essential for effectively implementing decentralized, people-centered models of participatory antimicrobial stewardship like ALKC. This requires the coordination and cooperation of all One Health stakeholders at the challenging ground level. It took Kerala four years to map all multi-sectoral AMR challenges and draft a time-sensitive, target-specific monitoring and evaluation framework.

## Conclusion

ASHI and participatory stewardship represent a transformative approach to combating antibiotic resistance. Through government-led initiatives focusing on policy, funding, regulation, and education, this system can significantly improve antibiotic use and safeguard public health. As the healthcare landscape continues to evolve, the widespread adoption of Antibiotic Smart Hospitals and participatory stewardship will be crucial in the global fight against antibiotic resistance. Kerala’s Antibiotic Smart Hospital initiative is a pragmatic, customized stewardship model that can address AMR challenges at different tiers of health care, especially in LMICs. Kerala Antibiotic Literate Campaign is a genuinely decentralized One Health approach to tackle AMR’s menace, highlighting the importance of political and administrative will in AMR action plans. When policymakers are convinced about the AMR crisis, it is easier for technical experts to conceptualize and execute AMR mitigation efforts!
